# Loss of Telomere Protection: Consequences and Opportunities

**DOI:** 10.3389/fonc.2013.00088

**Published:** 2013-04-15

**Authors:** Jacqueline J. L. Jacobs

**Affiliations:** ^1^Division of Molecular Oncology, The Netherlands Cancer InstituteAmsterdam, Netherlands

**Keywords:** telomeres, DNA-damage, genome instability, cancer, therapy

## Abstract

Telomeres are repetitive sequences at the natural ends of linear eukaryotic chromosomes that protect these from recognition as chromosome breaks. Their ability to do so critically depends on the binding of sufficient quantities of functional shelterin, a six-unit protein complex with specific and crucial roles in telomere maintenance and function. Insufficient telomere length, leading to insufficient concentration of shelterin at chromosome ends, or otherwise crippled shelterin function, causes telomere deprotection. While contributing to aging-related pathologies, loss of telomere protection can act as a barrier to tumorigenesis, as dysfunctional telomeres activate DNA-damage-like checkpoint responses that halt cell proliferation or trigger cell death. In addition, dysfunctional telomeres affect cancer development and progression by being a source of genomic instability. Reviewed here are the different approaches that are being undertaken to investigate the mammalian cellular response to telomere dysfunction and its consequences for cancer. Furthermore, it is discussed how current and future knowledge about the mechanisms underlying telomere damage responses might be applied for diagnostic purposes or therapeutic intervention.

## Introduction

Linear eukaryotic chromosomes pose multiple challenges to cells that need to be properly taken care of to avoid loss of proliferative capacity and genome integrity. As conventional DNA-polymerases cannot replicate the very ends of chromosomes, each cell division chromosome ends lose a bit of DNA-sequence, potentially causing loss of genetic information. Furthermore, cells contain multiple DNA-damage recognition and repair activities that act on exposed DNA-ends to halt cell cycle progression and fix chromosome-internal DNA-breaks to prevent unequal separation of genetic information into daughter cells. If these activities would continuously act on natural chromosome ends, cells would not be able to divide or properly segregate chromosomes during mitosis. Telomeres are specialized nucleoprotein structures at chromosome ends that cope with these challenges (de Lange, 2009; O’Sullivan and Karlseder, 2010). By consisting of long stretches of TTAGGG DNA-repeats and lacking genes, telomeres represent buffers that prevent replication-associated sequence loss at chromosome termini from reaching nearby genes. Moreover, telomeres protect natural chromosome ends from being recognized and processed as damaged DNA. For this mammalian telomeres rely on binding shelterin, a unique set of proteins composed of TRF1, TRF2, RAP1, TIN2, TPP1, and POT1, and on specific structural features. Telomeres contain a 3′ G-rich single-strand overhang that binds POT1, folds back and invades the duplex telomere repeat array. This forms a loop (t-loop) that presumably hides the extreme end of the chromosome from multiple activities.

**Figure 1:**
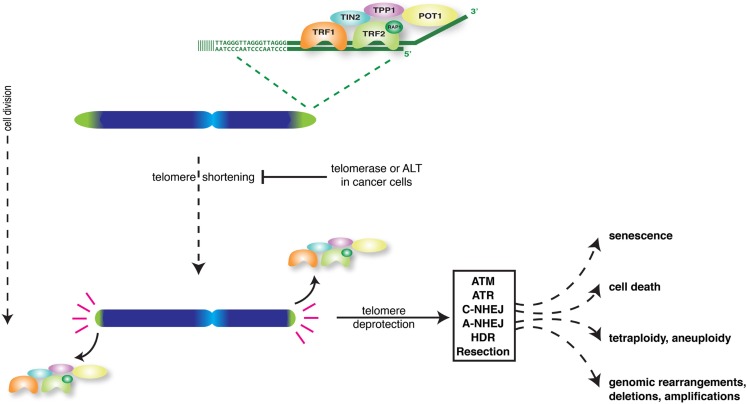
**Overview of the major consequences of loss of telomere protection in mammalian cells**.

While acting as buffers to prevent loss of genetic information, telomeres themselves shorten with every round of replication (Harley et al., 1990). Progressive shortening eventually causes telomeres to lose their protective activity, even before all telomere repeats are lost (Figure 1). Dividing cells can only avoid telomere deprotection when able to induce sufficient activity of the telomerase enzyme to add telomere repeats (Bodnar et al., 1998). Alternatively cells can engage the ALT mechanism of “alternative lengthening of telomeres” to lengthen telomeres through recombination (Cesare and Reddel, 2010). Normal human somatic cells do not have sufficient telomerase or ALT activity, which confronts them with short dysfunctional telomeres after a certain number of cell divisions.

Loss of telomere protection activates a DNA-damage-like signaling response that through induction of tumor suppressors p53 and p16 forces cells into senescence or apoptosis (d’Adda di Fagagna et al., 2003; Takai et al., 2003; Jacobs and de Lange, 2004, 2005; de Lange, 2010; O’Sullivan and Karlseder, 2010). This serves as an important tumor suppressor mechanism as it prevents the outgrowth of potentially cancerous cells. However telomere dysfunction can also be a source of genomic instability and put cells at risk of developing into cancer (Artandi and DePinho, 2010; Martinez and Blasco, 2010; Davoli and de Lange, 2011; Shay and Wright, 2011). This because DNA-repair activities at deprotected chromosome ends generate chromosome end-to-end fusions. In cells that escape senescence or apoptosis and divide, such fusions initiate breakage-fusion-bridge cycles that generate complex unbalanced chromosome rearrangements (Murnane, 2012). In addition, telomere dysfunction can lead to tetraploidization and chromosome mis-segregation (Davoli et al., 2010; Davoli and de Lange, 2012). Thus, depending on the effectiveness of the DNA-damage checkpoint in arresting or eliminating cells, telomere dysfunction either inhibits or promotes the development of cancer.

## Understanding the Consequences of Telomere Deprotection

**Figure 2:**
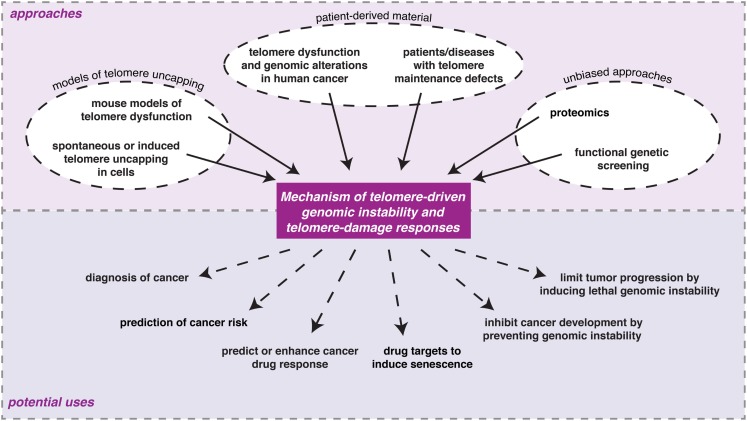
**Examples of the different experimental approaches being undertaken to understand the consequences of loss of telomere protection in mammalian cells and the potential ways in which information from such studies might eventually be used in the clinic**.

Multiple approaches have contributed to our current understanding of the molecular events and consequences associated with loss of telomere protection (Figure 2). These can roughly be grouped into three categories: (1) models of telomere uncapping in mammalian cells or mice; (2) analyses on patient-derived material; (3) unbiased and genome-wide approaches.

### Models of telomere uncapping in mammalian cells or mice

Telomere uncapping occurs when telomeres become critically short upon continued proliferation in the absence of telomerase. Telomere dysfunction is therefore ideally studied in a replicative senescence model where primary human cells are propagated *in vitro* until they reach their maximum lifespan, the so-called Hayflick limit, and stop proliferating. Analysis of the molecular changes induced at replicative senescence has yielded critical insights, such as that critically short telomeres are recognized by DNA-damage response (DDR) proteins (d’Adda di Fagagna et al., 2003). Despite the advantage of following the natural course of telomere uncapping, this approach also has limitations. One problem is that prolonged culturing of cells *in vitro* is associated with culture stress inducing complex telomere-independent cellular responses that partially overlap with telomere-mediated responses. Equally important, telomere uncapping due to shortening is not a synchronous process, complicating studies on telomere-dependent effects in a population of cells. Only a subset of cells experiences critically short telomeres at a given time and one has to wait until the majority of cells has senesced. This precludes detection of immediate effects of telomere uncapping. Studies on natural telomere deprotection in the context of an entire organism face additional challenges. These are caused by the variability in telomere lengths between individuals and between individual cells, but also by the slow speed of telomere shortening. A particular problem arises with the use of inbred laboratory mouse strains. While being a tremendously useful model system to study many different biological pathways, laboratory mice are not a good model to study the consequences of natural telomere shortening because, unlike humans, commonly used laboratory mouse strains have extremely long telomeres and high telomerase activity in all cells. During their normal lifespan such mice do not experience significant telomere uncapping (Blasco et al., 1997). Importantly, this also implies that most studies in laboratory mice, including those modeling cancer, do not incorporate the contribution of a telomere dysfunction component that would apply to humans.

A solution to problems associated with studying natural telomere shortening came from strategies in which telomere dysfunction is experimentally induced. Following the identification of the telomerase reverse transcriptase and RNA components and the different shelterin factors, significant knowledge about their function has come from experimental manipulation of telomerase or shelterin in tissue culture cells and mice. Apart from proving that telomeres control replicative lifespan and affect the development of cancer and aging-related pathologies, such studies also revealed many underlying molecular details (Artandi and DePinho, 2010; Martinez and Blasco, 2010; Sahin and Depinho, 2010; Shay and Wright, 2011; Tumpel and Rudolph, 2012). We now know that telomere function depends on both unique and redundant roles of shelterin components in protecting chromosome termini against six major threats: ATM-kinase activation, ATR-kinase activation, DNA-Ligase 4- and Ku70/80-dependent classical non-homologous end-joining (c-NHEJ), DNA-Ligase 3- and PARP-dependent alternative NHEJ (a-NHEJ), homologous recombination (HR), and end-resection (Figure 1) (Sfeir and de Lange, 2012). Activation of ATM and ATR results in checkpoint activation and proliferation arrest or apoptosis. c-NHEJ generates chromosome-end-to-end fusions without apparent major end-processing, leaving significant amounts of telomere repeats at the fusion sites. On the other hand, a-NHEJ, the primary pathway causing chromosomal translocations, uses microhomology and results in chromosome-end-to-end fusion with significant telomeric and subtelomeric deletion. Resection and HR both threaten telomere integrity, the latter for instance via unequal exchanges between sister telomeres that change telomere length.

Of the six threats, ATM and c-NHEJ are specifically blocked by TRF2, ATR is inhibited by POT1, while HR is inhibited by either POT1 or RAP1, and on top by Ku70/80. On the other hand, repression of a-NHEJ and end-resection depend on redundant functions of shelterin and in addition are repressed by Ku70/80 and 53BP1, respectively. While the TTAGGG-repeats provided by telomerase are needed to concentrate enough shelterin at chromosome ends, telomerase activity itself is regulated by shelterin, as well as by other factors and processes, together contributing to complex control of telomere maintenance (Cifuentes-Rojas and Shippen, 2012).

Studies in mice with manipulated shelterin or telomerase have shown the consequences of telomere deprotection on an organismal level. These studies clearly illustrate the opposite effects dysfunctional telomeres can have on the development of cancer, depending on the DNA-damage checkpoint status and stage of tumor development. Indeed, short telomeres in late-generation telomerase-deficient mice protect against cancer, while predisposing to aging (Greenberg et al., 1999; Rudolph et al., 1999). That is, when DNA-damage checkpoint activity by p53 is intact. In a p53-deficient background, where cells escape senescence or apoptosis, short telomeres accelerate the onset of epithelial tumorigenesis through increased genomic instability (Artandi et al., 2000). While telomere-driven genomic instability can promote cancer initiation, it can limit further cancer progression. Re-establishment of telomere protection by activation of telomerase or ALT facilitates further malignant progression by genome stabilization and improved cell viability (Begus-Nahrmann et al., 2012; Ding et al., 2012; Hu et al., 2012).

### Analyses on patient-derived material

Analyses of human cancers revealed that telomere shortening is widespread during human tumorigenesis. In addition, it was found that during their development all human tumors activate mechanisms to counteract further telomere shortening. Most tumors activate telomerase while 10–15% of tumors activate ALT (Bryan et al., 1997; Shay and Bacchetti, 1997). There is mounting evidence that concomitant with shortening, telomeres become dysfunctional during human cancer development (Artandi and DePinho, 2010). For example, anaphase bridges, a hallmark of dysfunctional telomeres leading to genomic instability, become more abundant at (pre)-invasive stages of colon and breast cancer. Moreover, in breast cancer the presence of short telomeres coincides with a sudden rise in genomic instability at the transition from ductal hyperplasia to carcinoma *in situ* and before the activation of telomerase (Chin et al., 2004). This, together with the recent PCR-based detection of telomere fusions in early breast cancer, provides strong evidence for the occurrence of telomere crisis during breast cancer development (Tanaka et al., 2012). In addition, syndromes associated with telomere replication defects, such as dyskeratosis congenita and Werner syndrome, are characterized by short telomeres, genomic instability, and an elevated incidence of spontaneous cancer (Armanios and Blackburn, 2012). Similarly, diseases accompanied by increased cell turnover, such as ulcerative colitis or liver cirrhosis, are associated with accelerated telomere shortening, telomere dysfunction, and cancer-predisposition. However, the currently most convincing data supporting a role for telomere dysfunction in cancer development stems from the molecular analysis of chronic lymphocytic leukemia (CLL) (Lin et al., 2010). A major advantage is that this cancer allows analysis of samples from patients at different stages of the disease. Single-molecule telomere length and telomere fusion analysis showed that short telomeres and fusions increase with more advanced disease, but are already present in a subset of patients prior to disease progression. Specifically patients with dysfunctional telomeres, not those with longer telomeres, were found to have large-scale genomic rearrangements concentrated in telomeric regions. Together the data strongly support the model that telomere attrition and fusion contribute to the progression of CLL. Moreover, the recent discovery of frequent POT1 mutations and associated telomeric and chromosomal abnormalities in CLL further reinforces the involvement of telomere dysfunction in the progression of this cancer (Ramsay et al., 2013).

Despite all analyses on human tumors the relative contribution of telomere deprotection to cancer development remains unclear. While next-generation sequencing approaches rapidly generate large amounts of data on (epi)genetic and transcriptional aberrations in human cancers, such approaches cannot easily reveal how many tumors developed under facilitation by telomere-driven genomic instability. For one, telomere repeats are filtered out during the analysis of such sequencing data, as they cannot be mapped to specific chromosomes. Also, current PCR-based methods do not detect all telomere fusions. Furthermore, telomere-driven genomic instability follows a hit-and-run mode. One single fusion of an uncapped telomere to another uncapped telomere or a DNA-double-strand break is sufficient to initiate breakage-fusion-bridge cycles where subsequent breaks and fusions do not necessarily involve telomeres but involve the entire genome. Thus both quantitative assessment of telomere fusion events and evaluation of the full extent of genomic alterations triggered by telomere uncapping, as opposed to other causes, remain difficult.

### Unbiased and genome-wide approaches

Biochemical fishing expeditions using mass-spectrometry or yeast-two-hybrid screens have tremendously contributed to our current knowledge about telomere biology by identifying multiple factors that interact with mammalian telomeres. These consist of shelterin components as well as many telomere-associated proteins that are not considered as part of shelterin because they have main functions outside of telomeres (Palm and de Lange, 2008; Dejardin and Kingston, 2009; Nittis et al., 2010). Among telomere-associated factors are nucleases, helicases, DNA-replication proteins, DDR factors, and the CST (CTC1, STN1, and TEN1) complex, which plays critical roles in controlling telomeric G-overhang length, telomere replication, and telomerase activity (Palm and de Lange, 2008; Chen et al., 2012; Gu et al., 2012; Sfeir, 2012; Wu et al., 2012). The finding that DDR factors are already present at telomeres that are sufficiently long to prevent activation of DNA-damage signaling responses was surprising. Subsequent work revealed that some of these factors in fact play protective roles. For instance Ku assists in protecting telomeres against HR and a-NHEJ, whereas ATM and ATR have been proposed to facilitate completion of telomere replication and formation of a proper telomere structure (Verdun et al., 2005; Celli et al., 2006; Verdun and Karlseder, 2006; Sfeir and de Lange, 2012).

Not all telomere-associated factors interact with telomeres constantly. Some interactions are restricted to certain cell cycle phases or specific conditions. For instance, when telomeres become critically short, some protein interactions disappear whereas new proteins now intensively associate with telomeres, such as 53BP1 and other DDR components. Likewise, differences exist between proteins present at telomeres maintained by telomerase versus those present at telomeres maintained by ALT. If proteomics-based approaches would be applied to compare different telomere states, such as capped versus uncapped, this could significantly increase our understanding of the mechanisms underlying control of telomere damage responses. A major challenge in such studies is that many signaling regulators are low in abundance or might only transiently or weakly associate with telomeres. Conventional immunoprecipitation and mass-spectrometry might therefore have to be complemented with more sensitive techniques, such as bimolecular fluorescent complementation that enables analysis of protein interactions in living cells and has recently been applied to identify proteins interacting with shelterin (Lee et al., 2011).

A powerful approach that proved very successful to identify key components of multiple cellular pathways, including the response to DNA-double-strand breaks, is gain-of-function or loss-of-function genetic screening in mammalian cells (Jacobs et al., 2000; Brummelkamp et al., 2004; Kolas et al., 2007; Paulsen et al., 2009; Ashworth and Bernards, 2010; Hurov et al., 2010; Cotta-Ramusino et al., 2011; Gudjonsson et al., 2012; Nguyen et al., 2012). Functional genetic screening has also proven powerful to identify novel telomere regulators in lower organisms, leading for instance to implication of the yeast KEOPs complex in telomere regulation (Downey et al., 2006). Since recently, functional genetic screening is also being applied in the field of mammalian telomere biology. For instance, RNA-interference screens have been initiated to identify factors involved in regulation of telomerase activity (Coussens et al., 2010; Cerone et al., 2011), ALT (Osterwald et al., 2012), telomere protection (Lackner et al., 2011), or telomere-driven genomic instability (Jacobs et al., unpublished). As an example, high-throughput RNA-interference screening identified multiple kinases, including ERK8, with no previous association to telomere biology as novel regulators of telomerase activity and attractive new drug targets for cancer therapy (Cerone et al., 2011). Given the previous successes in multiple experimental settings, recently initiated or future unbiased functional genetic screening approaches in mammalian systems hold significant potential for identifying new factors with important roles in the control of cancer or aging by telomeres.

## Opportunities

### Telomerase-based approaches

The strong dependence on telomere maintenance for sustained tumor growth and the presence of telomerase in 90% of cancers, but not most somatic cells, have inspired development of telomerase-based strategies to inhibit cancer cell growth and triggered evaluation of telomerase as a biomarker or prognostic marker for cancer (Ruden and Puri, 2012; Mocellin et al., 2013). Multiple telomerase-based strategies are currently evaluated in clinical trials, but it is still unclear how beneficial such strategies are to cancer patients. This is in part due to incomplete understanding of the effects of inhibiting telomerase on both cancer cells and normal cells. Telomerase is important for the renewal capacity of normal stem and progenitor cells, which could cause unwanted side-effects. Telomerase has also been implicated in telomere-independent pathways, making the outcome of its inhibition less predictable until these pathways and roles of telomerase therein are fully understood (Martinez and Blasco, 2011; Ghosh et al., 2012). In addition, as telomere uncapping can cause genomic instability, there is a risk that telomerase inhibition might in fact accelerate progression of some tumor cells (Pereira and Ferreira, 2013). Moreover, dysfunctional telomeres cause impaired mitochondrial function and promote oxidative stress, which might lead to accumulation of additional mutations that promote tumor progression (Sahin et al., 2011). Furthermore, the generation of senescent cells by telomere uncapping to limit outgrowth of the targeted tumor, comes at the cost of generating a permissive environment for tumor progression. Namely, senescent cells are known to secrete multiple factors that can promote tumor growth and invasion of surrounding (pre-)malignant cells (Rodier and Campisi, 2011). The risks associated with telomerase inhibition and telomere uncapping might be less of a concern in patients with advanced cancer, but they could limit the use of telomerase-based strategies in young patients or patients with early disease.

### Alternative strategies

Understanding the molecular mechanisms underlying telomere protection, telomere-driven senescence and genomic instability will not only increase predictability and understanding of the efficacy of telomerase-based strategies, but will also facilitate development of novel therapeutic strategies for cancer and (premature) aging-related disease (Figure 2). For cancer these could aim to provoke senescence, as with telomerase inhibition. However, particularly interesting would be to think of alternative strategies that avoid unwanted side-effects or risks of existing approaches, such as associated with induction of senescence. A potential, but challenging strategy could be to inhibit telomere-driven genomic instability before obvious cancer presentation to prevent or slow down cancer development. This might for instance be applied to people predisposed to cancer because of a condition associated with accelerated telomere shortening. Inhibition of telomere-driven genomic instability could be achieved by inhibiting telomere fusion by interfering with DNA-repair activities at uncapped telomeres. Such a strategy should not impair regular error-free DNA-repair at other sites, to avoid increasing cancer risk. An intriguing opposite strategy might in fact be to increase genomic instability such that cancer cells die of crisis, by “tipping the balance.” High levels of genomic instability, that may drive initiation of early lesions, need to be reduced to allow efficient tumor progression. Nevertheless, many cancers are hallmarked by an instable genome, hyper-sensitizing them to genome-destabilizing strategies. Interestingly, increased and ongoing chromosomal instability is also common for tumors utilizing ALT, along with telomeres that are recognized as DNA-damage, but appear not to fuse (Scheel et al., 2001; Cesare et al., 2009; Lovejoy et al., 2012). Besides interfering with the ALT mechanism, alleviating repression of telomere fusion might be an effective way to kill ALT tumor cells by inducing rampant genomic instability. This is an especially intriguing possibility given that ALT tumors do not rely on telomerase for telomere maintenance and would thus be resistant to telomerase-based therapeutics.

The development and adequate application of such new therapeutic strategies, require thorough investigation and understanding of the mechanisms underlying control of DDR and repair activities at telomeres and DNA-lesions. Apart from revealing new drug targets, studies addressing this might also contribute to prediction of therapy responses to DNA-damage inducing anti-cancer drugs or to development of strategies to enhance cancer drug sensitivity. With respect to the latter, it is interesting to note that dysfunctional telomeres have been shown to increase the sensitivity of cells to ionizing radiation and DNA-damaging chemotherapeutics (Wong et al., 2000; Lee et al., 2001; Soler et al., 2009). In addition, insights into the precise events associated with telomere uncapping could contribute to the establishment of diagnostic tools, such as for early detection of cancers and identification of cancer-predisposed individuals.

## Conflict of Interest Statement

The authors declare that the research was conducted in the absence of any commercial or financial relationships that could be construed as a potential conflict of interest.
